# Nanocarriers containing platinum compounds for combination chemotherapy

**DOI:** 10.3389/fphar.2022.1050928

**Published:** 2022-11-08

**Authors:** Guihua Fang, Aiwen Zhang, Lu Zhu, Qiuxiang Wang, Feilong Sun, Bo Tang

**Affiliations:** School of Pharmacy, Nantong University, Nantong, Jiangsu, China

**Keywords:** platinum compounds, side effects, resistance, nanocarriers, combination chemotherapy

## Abstract

Platinum compounds-based drugs are used widely in the clinic for the treatment of many types of cancer. However, serious undesirable side effects and intrinsic or acquired resistance limit their successful clinic use. Nanocarrier-based combination chemotherapy is considered to be an effective strategy to resolve these challenges. This review introduces the recent advance in nanocarriers containing platinum compounds for combination cancer chemotherapy, including liposomes, polymer nanoparticles, polymer micelles, mesoporous silica nanoparticles, carbon nanohors, polymer-caged nanobins, carbon nanotube, nanostructured lipid carriers, solid lipid nanoparticles, and multilayered fiber mats in detail.

## Introduction

**FIGURE 1:**
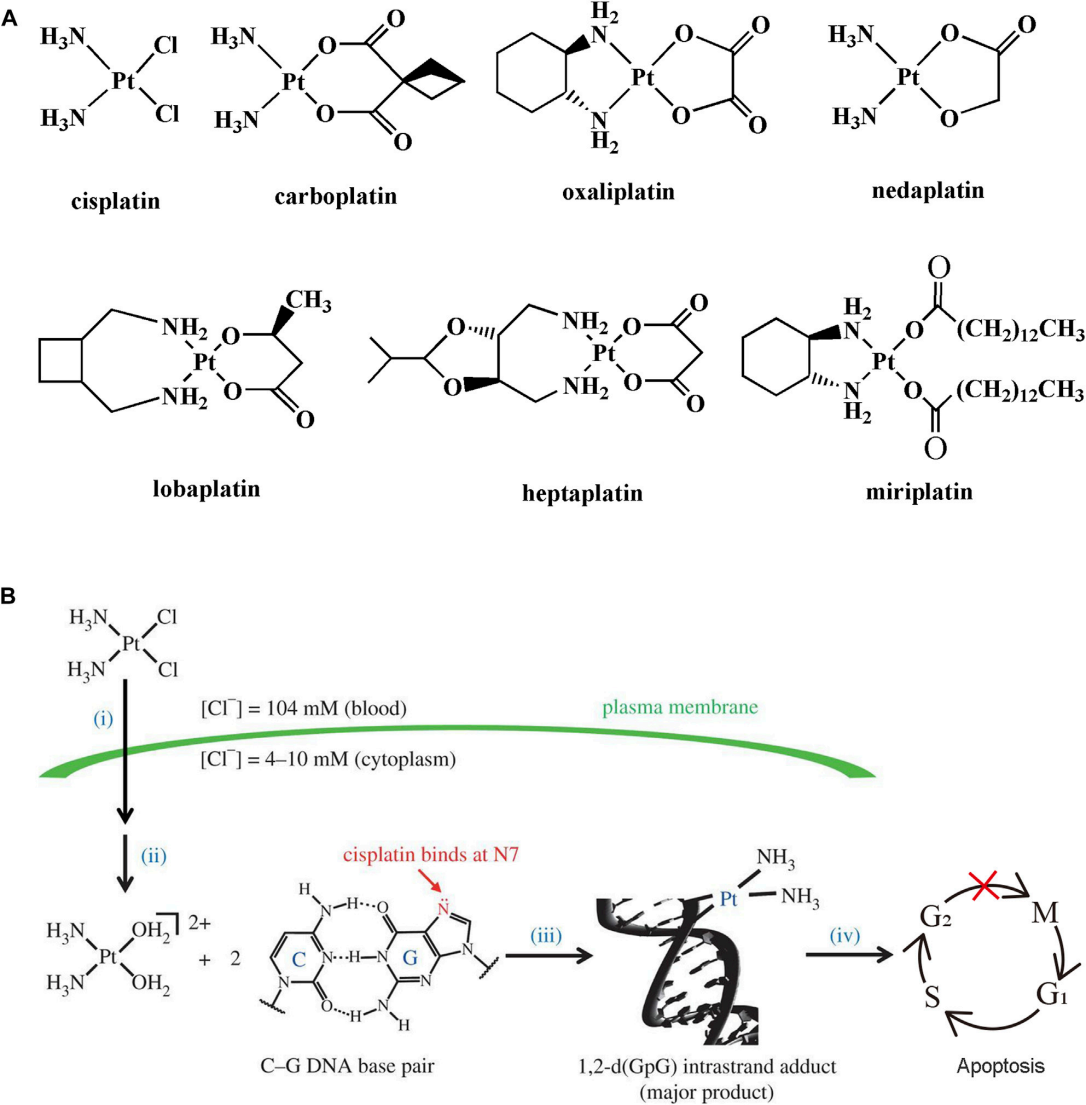
**(A)** Chemical structures of clinically used platinum-based anticancer drugs. **(B)** Mechanism action of cisplatin. (i) Cellular uptake, (ii) activation, (iii) DNA binding, and (iv) DNA damage trigger cell apoptosis. Reproduced from reference (Johnstone et al., 2015) with permission.

Cancer is a primary cause of death and a significant obstacle to prolonging life spans around the world. According to GLOBOCAN 2020, the estimated number of new cases and deaths from cancer was 19.3 and 10 million, respectively (Sung et al., 2021). Platinum compounds-based drugs are used widely in the clinic for the treatment of various types of cancer, such as lung, testicular, gastric, bladder, ovary, and colorectal (Dilruba and Kalayda, 2016). Cisplatin, the first platinum compound, was prepared by Italian chemist Michel Peyrone in 1844 (Kauffman et al., 2010). Over one hundred years later, Rosenberg and his group found that the electrolyte of platinum electrode had an inhibitory effect on the cell division of *Escherichia* Coli (Rosenberg et al., 1965). It is inferred that platinum compounds can inhibit the growth of bacteria. Then, they discovered the anticancer effect of cisplatin in mice bearing sarcoma 180 and leukemia L1210 in 1969 (Rosenberg et al., 1969). Cisplatin was approved for the treatment of testicular and ovarian cancer by the US FDA in 1978 (Ghosh 2019). Cisplatin represents a success story in inorganic medicinal chemistry. It initiates the innovation in the development of platinum-based anticancer drugs until today. Currently, seven platinum-based anticancer drugs are approved for clinic use, including cisplatin, carboplatin, oxaliplatin, nedaplatin, lobaplatin, heptaplatin, and miriplatin (Johnstone et al., 2016) (Figure 1A).

The mechanism of action of cisplatin and related platinum compounds has been elucidated over the past few decades (Wang and Lippard, 2005; Johnstone et al., 2015) (Figure 1B). The generally accepted mechanism contains the following four steps: 1) the dissolved cisplatin travels through the bloodstream to the extracellular matrix. The cellar uptake of cisplatin is mediated by passive diffusion or active transport relied on the membrane protein; 2) the concentration of chloride ions is uneven distribution inside and outside of the cells. The intracellular chloride ion concentration (4–10 mM) is much lower than that of the extracellular matrix (104 mM), which is beneficial for transformation of cisplatin to its cation hydrate such as cis-[Pt(NH_3_)_2_(OH_2_)_2_]^2+^; 3) platinum hydrate with strong electrophilic tendency preferentially binds to the N7 position of guanosine on DNA. The adjacent DNA bases are crosslinked by platinum hydrate; 4) finally, the DNA damage triggers cell cycle arrest at the S/G2 stage and induces cell death by apoptosis if the damage cannot be repaired.

Although platinum-based drugs have been widely used in the clinic to treat several types of cancers, many serious undesirable side effects and intrinsic or acquired resistance limit the further clinic use of platinum-based drugs (Markman 2003; Oun et al., 2018; Zhou et al., 2020). Common side effects include nephrotoxicity, myelosuppression, neurotoxicity, anaphylaxis, cytopenias, hepatotoxicity, ototoxicity, cardiotoxicity, and so on (Wong and Giandomenico, 1999; Mjos and Orvig, 2014; Oun et al., 2018). The various side effects prevented the administration of higher drug dosage. To relieve the side effects, patients need to lower the administration dosage. Moreover, patients need careful monitoring of their functional and biochemical markers during treatment and taking other non-chemotherapeutic drugs to combat side effects, such as antiemetics, propafenone, and antioxidants (Oun et al., 2018). The resistance mechanism of platinum-based drugs is badly complicated. It mainly includes decreased intracellular drug concentration, DNA damage and repair, evading apoptosis and autophagy (Ohmichi et al., 2005; Zhou et al., 2020). Acquired resistance to platinum drugs is mainly associated with a dramatic reduction of drug accumulation in the cell. Current studies have indicated that in addition to passive diffusion, the cellular uptake of platinum drugs was also medicated by multiple transporters, such as organic cation transporters (OCTs), copper transporters (CPTs) and multidrug resistance protein (MRP) (Ohmichi et al., 2005; Yokoo et al., 2007; Howell et al., 2010). Disease conditions can affect transporter expression, and thereby altering the cellular uptake of platinum drugs and affecting the therapeutic efficacy. Moreover, the enhanced DNA repair process exerted a tremendous influence on drug resistance (Howell et al., 2010). The mechanisms of DNA damage repair are complex. A nucleotide excision repair system could excise the damaged nucleotides in the majority of intrastrand cross-links and reconstruct the gene integrity (Yokoo et al., 2007). The function of many components played nonnegligible roles in the DNA repair process, such as breast cancer susceptibility genes and excision repair cross-complementing members (Oberoi et al., 2013; Zhang et al., 2022). Furthermore, evading apoptosis is a universal phenomenon of cancer cells (Parhi et al., 2012). To evade apoptosis, platinum-resistant cancer cells often overexpress anti-apoptotic proteins (e.g., Bcl-2) (Qin et al., 2018). It was also reported that pro-survival signal pathway (e.g., PI3K/AKT/mTOR pathway), epigenetic change, and tumor microenvironment also contributed to the development of drug resistance (Hu et al., 2016; Prihantono and Faruk, 2021; Pomeroy et al., 2022). In addition, drug-induced autophagy increased in the platinum-resistant cells after treatment with platinum drugs, and the addition of autophagy inhibitors could effectively reduce drug resistance (Lei et al., 2019). However, some scientists have come to the opposite conclusion (Li et al., 2014; Mokhtari et al., 2017). Therefore, further studies on the relationship between autophagy and platinum resistance are warranted.

Currently, numerous strategies have been explored to reduce the side effects and resistance of platinum drugs, such as chemical modification of the platinum drugs with a targeting moiety, delivery of platinum drugs using nanomaterials or hydrogels, combination chemotherapy, preparation of platinum nanoclusters and glutathione-scavenging platinum drugs (Oberoi et al., 2013; Oun et al., 2018; Zhang et al., 2022). Among them, combination chemotherapy and nanotechnology have received a lot of attention (Parhi et al., 2012). Combination chemotherapy has been widely used in clinics to solve the problems caused by single cancer chemotherapy. Combination chemotherapy involves the application of two or more drugs or simultaneous use of different therapeutic methods such as chemotherapy, ultrasound therapy, electrotherapy, and radiotherapy (Hu et al., 2016; Qin et al., 2018; Pomeroy et al., 2022). In these methods, the most common combination therapeutic modality is the co-administration of different therapeutic drugs. Combination chemotherapy can allow each drug to exert anticancer effects at a lower dosage by synergistically targeting different signal pathways or molecular targets, which could overcome resistance mechanism and reduce the side effects associated with the high dose of a single-drug, or retard the cancer adaptation through regulating the genetic barriers (Mokhtari et al., 2017; Lei et al., 2019; Prihantono and Faruk, 2021). Furthermore, combination chemotherapy can allow drugs synergistically target the same cellular signal pathways or molecular targets, improving the therapeutic effect (Li et al., 2014; Mokhtari et al., 2017). Dasanu et al. reported that the combined administration of carboplatin and gemcitabine was a safe and effective therapeutic method for metastatic ovarian cancer with reduced hematological toxicity (Dasanu et al., 2009).

Although combination administration has made some extent progress in the treatment of cancer, the huge achievement is impeded by poor water solubility, insufficient drug accumulation in the tumor tissue, rapid drug elimination, and improper *in vivo* distribution (Greco and Vicent, 2009). To overcome these limitations aforementioned, it is urgent to develop suitable drug delivery systems that could optimize the *in vitro* and *in vivo* characteristics of drugs and reduce the toxic and side effects. Nanotechnology has been proposed as an effective approach to improving the therapeutic effect of various cancers. Nanocarriers could increase the water solubility of poorly soluble drugs, prolong blood circulation time, and endow drugs with tumor targetability, which enhances the availability of drugs in the tumor cell while reducing the toxic and side effects associated with conventional chemotherapy (Bertrand and Leroux, 2012; Moradi Kashkooli et al., 2020). Several nanocarriers for cisplatin or cisplatin combination therapy have entered clinical trials. Lipoplatin, a liposomal cisplatin formulation with a particle size of about 110 nm, has passed phase III clinical trials, which exhibited excellent anticancer efficacy in several tumors, such as lung, colon, gastric, and prostate cancers (Li et al., 2015). In addition, Poly(glutamic acid) (PGlu) has been used widely in drug delivery systems. NC-6004 was a PGlu-based polymeric micelles containing cisplatin. It reduced nephrotoxicity compared with cisplatin alone in the clinical trials for the treatment of head and neck tumors, and pancreatic cancer (Song et al., 2014; Shen et al., 2017). Then, NC-6004/gemcitabine combination increased the median overall survival rate in a phase II study for the treatment of pancreatic cancer (He et al., 2015). These clinical trials demonstrated that nanocarriers containing platinum drugs have a promising prospect for the treatment of cancers. In this review, we provide a focused review on the use of nanocarriers to synergistically deliver platinum compounds and others for cancer therapy.

## Nanocarriers containing platinum compounds for combination chemotherapy

Nanocarriers have been demonstrated to be effective drug carriers that can deliver two or more drugs with different physicochemical properties and pharmacological action and mechanisms for the treatment of cancer. The application of nanocarriers to encapsulate platinum compounds and other drugs for combination chemotherapy has been extensively studied and the details are summarized in Table 1. Cisplatin is the earliest and most widely used platinum compound for the treatment of various cancers in the clinic, such as ovaries, testes, head and neck, and lung cancer. However, unwanted side effects and drug resistance limited its more successful clinical application. Combination chemotherapy is a relatively effective method to reduce the unwanted side effects and drug resistance of cisplatin (Yang et al., 2017). Cisplatin-based combination chemotherapy has obtained some progress in the clinic. For example, cisplatin combined with etoposide or irinotecan for lung cancer; cisplatin combined with 5-fluorouracil, or gemcitabine, or paclitaxel for head and neck cancer (Gerweck, 2000; Chou, 2006; Liu et al., 2017; Yu et al., 2020). However, highly effective combination chemotherapy is not easy to design due to its different physicochemical and biological properties. Nanocarriers offered a better opportunity to address this problem. Among the combination chemotherapy of nanocarriers containing platinum compounds and other drugs, the combination of cisplatin and paclitaxel has received the most attention. Various nanocarriers have been prepared for the co-delivery of cisplatin and paclitaxel including mPEG-b-P(LA-co-MCC/Cis Pt(IV)) and mPEG P(LA-co-MCC/paclitaxel) self-assemble micelles (Xiao et al., 2012), telodendrimer micelles (Cai et al., 2015), mPEG-b-PAGE/MPA-b-PLA micelles (Li et al., 2015), mPEG-b-P(Glu)-b-P(Phe) micelles (Song et al., 2014), Pt(IV)-conjugated copolymer (mPEG-PLGA) micelles (Shen et al., 2017), Folic acid-modified PEG-PLGA nanoparticles and nanostructured lipid carriers (He et al., 2015; Yang et al., 2017), Trans-activating transcriptional activator modified solid lipid nanoparticles (Liu et al., 2017). Xuesi Chen’s Group prepared mPEG-b-P(Glu)-b-P(Phe) triblock copolymer self-assembled micelles and explored its co-delivery of cisplatin and paclitaxel for the treatment of lung cancer (Song et al., 2014). The formed core-shell-corona micelles had three specific zones: the inner hydrophobic P(Phe) core for loading paclitaxel, the anionic P(Glu) middle shell for cisplatin complexation, and PEG outer shell as protection corona. *In vitro* release experiments showed that paclitaxel released faster than cisplatin, which was due to the physical encapsulation of PTX compared to the complexation of Pt(II). Moreover, cisplatin exhibited fast-release behavior at pH 5.5 compared to that at pH 7.4, which was attributed to the weakened complexation of Pt(II) caused by the protonation of carboxylic groups of P(Glu) in an acidic medium. The fast-release behavior is beneficial for anti-tumor therapy because of the acidic microenvironment in tumor tissue (Gerweck, 2000). The cytotoxicity test showed that the proliferation inhibition effect of the double-drug loaded micelles was greater than that of the single-drug-loaded micelles only at 72 h, which was ascribed to the retarded drug release by double-drug loaded micelles. With the extension of time, the combined effect became more obvious. The combination index (CI) was often used to evaluate the synergistic effect (Chou, 2006). The double-drug loaded micelles showed a high synergistic effect on A549 cells in the range of drug effect levels (CI<1), while the free drug combination showed an obvious antagonistic effect (CI＞1). Finally, the *in vivo* anti-tumor efficacy and system toxicity were evaluated in Balb/C nude mice bearing xenograft A549 human lung tumor. Dual-drug-loaded micelles showed a better tumor suppression rate (83.1%) than that of single paclitaxel groups (58.1% for paclitaxel solution and 67.8% for paclitaxel micelles) and free drug combination group (73.8%). In addition, the dual-drug-loaded micelles did not cause serious weight loss during treatment compared to the single paclitaxel group and free drug combination group. Overall, dual-drug-loaded micelles offer a better therapeutic efficacy in comparison with single-drug-loaded micelles and free drug combination groups.

**TABLE 1:** Recent advances in nanocarriers containing platinum compounds and other drugs for combination chemotherapy.

Drugs	Drug delivery systems	Results	Reference
Cisplatin/paclitaxel	mPEG-b-P(LA-co-MCC/Cis Pt(IV)) and mPEG P(LA-co-MCC/paclitaxel) self-assemble micelles	(i) Micelles showed pH-sensitive drug release profiles	Xiao et al. (2012)
		(ii) The dual-drug-loaded micelles significantly reduced the IC50 than free paclitaxel, free cisplatin, and free drug combination against SKOV-3 cells	
		(iii) Micelles formulations exhibited comparable or even better anti-tumor effects and much lower systematic toxicity than free drug combination	
Cisplatin/paclitaxel	Telodendrimer micelles	(i) Telodendrimer micelles (Cisplatin/paclitaxel = 2) showed the best cell inhibition effect (lowest IC50) in SKOV-3, ES-2 cells, and Hela cells	Cai et al. (2015)
		(ii) The results of real-time fluorescence imaging and Pt biodistribution studies showed that micelles formulation had the highest tumor uptake of PTX and CDDP after i.v. injection in the nude mice bearing tumor, while reducing the drug accumulation in the liver, kidney, and spleen	
		(iii) Telodendrimer micelles (Cisplatin/paclitaxel = 2) showed the longest survival time among all groups in mice bearing SKOV-3 ovarian cancer	
Cisplatin/paclitaxel	mPEG-b-PAGE/MPA-b-PLA micelles	(i) Micelles formulation showed sustained release profiles	Li et al. (2015)
		(ii) Dual-drug-loaded micelles enhanced synergistic inhibition effect and relieved drug resistance in both SMMC-7721 and SMMC-7721 resistance cells compared with free drug alone and single-drug-loaded micelles	
Cisplatin/paclitaxel	mPEG-*b*-P(Glu)-*b*-P(Phe) micelles	(i) Cisplatin cross effect retarded paclitaxel release from the micelles	Song et al. (2014)
		(ii) Dual-drug-loaded micelles (paclitaxel/cisplatin = 0.3) showed the best tumor suppression rate (83.1%) in the treatment of xenograft human lung tumors than other groups	
		(iii) Micelles formulation showed no obvious damage to the liver and kidney compared to the free drug	
Cisplatin/paclitaxel	Pt(IV)-conjugated copolymer (mPEG-PLGA) micelles	(i) Pt(IV)-conjugated copolymer micelles showed a sol−gel conversion with elevating the temperature	Shen et al. (2017)
		(ii) The micelles showed controlled drug release profiles for 2.5 months	
		(iii) Dual-drug-loaded micelles showed an excellent anticancer effect in nude mice bearing human ovarian tumor and reduced side effects after a single intratumoral injections	
Cisplatin/paclitaxel	Folic acid-modified PEG-PLGA nanoparticles	(i) Nanoparticles significantly prolonged the blood circulation of free drug	He et al. (2015)
		(ii) Dual-drug-loaded micelles (paclitaxel/cisplatin = 2) showed the best anticancer effect in male Balb-c/nude mice xenografted A549 and M109 lung cancer	
Cisplatin/paclitaxel	Folate-decorated nanostructured lipid carriers(FA NLCs)	(i) IC50 value of dual-drug-loaded FA NLCs (0.6 ± 0.1 μM) was lower than free drug, single-drug-loaded NLCs, and dual-drug-loaded NLCs	Yang et al. (2017)
		(ii) the tumor growth was significantly retarded by dual-drug-loaded FA NLCs than other formulations	
α-tocopherol succinate -cisplatin/paclitaxel	Trans-activating transcriptional activator modified solid lipid nanoparticles (TAT SLNs)	(i) TAT SLNs significantly increased the cellular uptake than other SLNs in HeLa cells	Liu et al. (2017)
		(ii) Dual-drug-loaded TAT SLNs exhibited the highest tumor inhibition rate (72.2%) than that of unmodified SLNs (58.9%) in cervical cancer-bearing mice	
Cisplatin/doxorubicin	Hyaluronic acid micelles	(i) Dual-drug-loaded micelles showed higher cellular uptake and stronger cytotoxicity than free drug against 4T1 (CD44^+^) breast cancer cells	Yu et al. (2020)
		(ii) Dual-drug-loaded micelles showed a higher tumor inhibition rate (∼66%) than that of the free drug combination groups (∼50%)	
Cisplatin/doxorubicin	Poly(acrylic acid) modified mesoporous silica nanoparticles	(i) pH-responsive drug release: 70.0% of Pt(II) and 79.9% of doxorubicin were released within 144 h at pH 5.5, while only 15.9% of Pt(II) and 25.3% of doxorubicin was released at pH 7.4	Li et al. (2016)
		(ii) Cytotoxicity test: dual-drug-loaded nanoparticles showed lower cell viability in A357 (8.8%) and HeLa cells (25.6%) than that of a free drug and single-drug-loaded nanoparticles at 48 h	
Cisplatin/doxorubicin	Single-Walled Carbon Nanohorns (SWCNHs)	(i) The combination index (CI) of *in vitro* chemotherapeutic and chemo-photothermal synergistic antitumor effects was 0.439 and 0.396, respectively	Yang et al. (2018)
		(ii) Dual-drug-loaded SWCNHs showed a longer half-life (10.9 h) and could ablate primary breast tumors and lung metastases	
Cisplatin/doxorubicin	Platinum-Conjugated, Doxorubicin-Loaded Polymer-Caged Nanobins (Pt-PCN)	(i) Pt-PCN exhibited pH-responsive Pt-release profiles *in vitro*	Lee et al. (2010)
		(ii) Pt-PCN showed a lower combination index (CI) (0.34) compared with that of the free drug combination (2.08) in MDA-MB-231 human breast cancer cells	
Cisplatin/doxorubicin	c(RGDfK) modified multi-walled carbon nanotube (MWCNT)	Dual-drug-loaded c(RGDfK) modified MWCNT exhibited higher cytotoxicity than either free drug alone or free drug combination in A2780, A2780/Cis, and Ishikawa cells	Chin et al. (2014)
Cisplatin/doxorubicin/camptothecin	Nanoscopic brush-arm star polymers (BASPs)	Three-drug-loaded BASPs showed higher cytotoxicity than the one- and two-drug-loaded systems OVCAR3 cells	Liao et al. (2014)
Cisplatin/gemcitabin	Micelle-containing PEGylated liposomes	(i) Cisplatin-loaded PLG-PEG micelles and gemcitabine were co-encapsulated into PEGylated liposomes by the Thin Film Processing Method	Liu et al. (2021)
		(ii) *In vivo* pharmacokinetic studies indicated that dual-drug-loaded liposomes had higher AUC (2.63- and 45.76-folds) compared with cisplatin and gemcitabine	
		(iii) The tumor inhibition rate of dual-drug-loaded liposomes was 2.83-, 1.86-, and 1.58-folds higher than that of gemcitabine liposomes, cisplatin liposomes, and cisplatin/gemcitabine solution, respectively	
Cisplatin/curcumin	mPEG-SS-PBAE-PLGA nanoparticles	(i) Platinum curcumin complexes were encapsulated into pH and redox dual-responsive nanoparticles by a nano-precipitation method	Chen et al. (2020)
		(ii) Dual-drug-loaded dual-responsive nanoparticles showed 4-fold higher cytotoxicity than the free drug	
		(iii) *In vivo* pharmacodynamics demonstrated that nanoparticles enhanced the anticancer effect and anti-metastasis activity while reducing toxicity	
Cisplatin/demethylcantharidin	Dual-sensitive and dual-drug backboned shattering polymer nanoparticles	(i) Nanoparticles showed redox- and pH-responsive drug release	Cong et al. (2018)
		(ii) *In vivo* distribution and metabolism of NPs can be monitored by Pt-based drug-mediated computer tomography	
		(iii) Dual-drug-loaded nanoparticles could eradicate the tumor burden on a high-fidelity patient-derived lung cancer model	
Cisplatin/p62siRNA/β5 plasmid DNA	Nanoparticles consisted of (i) inner core: cisplatin loaded polylactic acid nanoparticles and (ii) outer layer: cationic chitosan (CS) linked P62 siRNA and β5 plasmid DNA	(i) Nanoparticles reduced the cisplatin resistance index (4.62) compared with free cisplatin (3.62) in the 2008/C13 cell	Babu et al. (2014)
		(ii) Triple drug-loaded nanoparticles significantly reduced the IC50 value in 2008/C13 cells compared to that of single- or dual-drug-loaded nanoparticles	
Carboplatin/paclitaxel	Crosslinked multilamellar liposomes (cMLVs)	*In vitro* and *in vivo* studies showed that the co-delivery of two drugs with cMLVs effectively inhibited the proliferation of ovarian cancer	Zhang et al. (2017)
Carboplatin/paclitaxel	PLGA-PEG nanoparticles	(i) The amino group of carboplatin was conjugated with the carboxylic group of PLGA-PEG-COOH. Then, the polymeric prodrug self-assembles to form nanoparticles for encapsulating paclitaxel	Zhang Z et al. (2016)
		(ii) Dual-drug-loaded nanoparticles showed the best tumor inhibition rate (79%) than other formulations	
Carboplatin/paclitaxel	Folic acid-PEG- conjugated *p*-phosphonated calyx[4]arene nanoparticles (Fp-PCN)	Fp-PCN inhibited ovarian cancer cell proliferation, apoptosis, and invasive capacity through the JMJD3-HER2 axis	Mo et al. (2017)
Oxaliplatin/dichloroacetate	Multilayered fiber mats (MFM)	(i) Dual-drug-loaded MFM showed a time-programmed drug release profile	Zhang W et al. (2016)
		(ii) Dual-drug-loaded MFM improved the anti-recurrence effect and reduced toxic effects over 30 days on a murine cervical carcinoma model compared with drug-loaded monolayered fiber mat	
Carboplatin/gemcitabine	Nanoscale coordination polymers (NCP)	The dual-drug-loaded NCP significant increased blood circulation time (11.8 ± 4.8 h) and drugs accumulation in tumor (10.2 ± 4.4 %ID/g at 24 h), leading to higher tumor growth inhibition (80%) in SKOV-3 and A2780/Pt-resistant tumors, respectively, compared with the control group	Poon et al. (2016)

Apart from the aforementioned co-delivery nanosystems for cisplatin and paclitaxel, co-delivery of cisplatin and doxorubicin by hyaluronic acid micelles, poly(acrylic acid) modified mesoporous silica nanoparticles, single-walled carbon nanohorns, polymer-caged nanobins, c(RGDfK) modified multi-walled carbon nanotube, and nanoscopic brush-arm star polymers has made significant progress in the treatment of cancers (Lee et al., 2010; Chin et al., 2014; Liao et al., 2014; Li et al., 2016; Yang et al., 2018; Yu et al., 2020). In addition to paclitaxel and doxorubicin, designed nanocarriers for the co-delivery of platinum drugs and gemcitabin, curcumin, demethylcantharidin, dichloroacetate, and gene drugs have also been extensively investigated (Babu et al., 2014; Poon et al., 2016; Zhang W et al., 2016; Cong et al., 2018; Chen et al., 2020; Liu et al., 2021). Therefore, nanocarriers containing platinum drugs and other drugs are a promising strategy for combination chemotherapy, which can prolong blood circulation time, increase drug accumulation in tumor cells, as well as enhance therapeutic efficacy, overcome intrinsic or acquired resistance and alleviate the toxic and side effects.

## Conclusion and perspectives

Platinum drugs have been used commonly for the treatment of cancers in clinics. However, harmful side effects and drug resistance limited it to yielding better therapeutic efficacy. Considerable efforts have been made to solve these thorny issues until the advent of nanotechnology, which brought much confidence to scientists. The application of nanocarriers for the co-delivery of platinum compounds and other drugs for combination chemotherapy has achieved quite a few progresses owing to its advantages in overcoming intrinsic or acquired resistance and reducing side effects caused by the treatment with platinum agents. To date, just a few nanocarriers containing platinum drugs have entered clinical trials for combination chemotherapy. Therefore, more innovative studies are needed to facilitate the translation of nanocarriers containing platinum compounds and other drugs to the clinical product. First, the physicochemical properties, action mechanisms, ratio, and dosage of multiple-drug can affect the *in vitro* release behavior, cell uptake, and biodistribution, so to obtain a better synergistic anticancer effect, the desired combined drugs, ratio, and dosage in nanocarriers need to be optimized through multiple tests *in vitro* and *in vivo*. Furthermore, the constructed nanocarriers should ensure the release of drugs in tumor tissues and cells, such as tumor-targeting nanocarriers and stimulus-responsive nanocarriers, thereby improving therapeutic efficacy and reducing toxic side effects. Despite the design of nanocarriers for combination chemotherapy is complicated, building more intelligent nanocarriers based on the pharmacological purposes of different anticancer drugs will make our dream come true.
